# Health-related quality of life in patients with sickle cell disease in Saudi Arabia

**DOI:** 10.1186/s12955-015-0380-8

**Published:** 2015-11-16

**Authors:** Anwar E. Ahmed, Ahmed S. Alaskar, Ahmad M. Al-Suliman, Abdul-Rahman Jazieh, Donna K. McClish, Majid Al Salamah, Yosra Z. Ali, Hafiz Malhan, May Anne Mendoza, Abdulrahman O. Gorashi, Mohamed E. El-toum, Wala E. El-toum

**Affiliations:** King Saud bin Abdulaziz University for Health Sciences, MC 2350, P.O. Box 22490, Riyadh, 11426 KSA; King Abdullah International Medical Research Center, Riyadh, Saudi Arabia; King Fahad Hospital, Hofuf, Saudi Arabia; King Abdulaziz Medical City, Riyadh, Saudi Arabia; Department of Biostatistics, Virginia Commonwealth University, Richmond, VA USA; King Fahad Central Hospital, Jazan, Saudi Arabia

**Keywords:** Sickle cell disease, Quality of life, SF-36, Pain, Saudi Arabia

## Abstract

**Background:**

There is a lack of research concerning health-related quality of life (HRQoL) in Saudi patients with sickle cell disease (SCD), particularly among adult populations. The aim of the current study was to describe the characteristics of SCD patients and their impact on their quality of life (QoL).

**Methods:**

Six hundred twenty-nine adult SCD patients who attended King Fahad Hospital in Hofuf and King Fahad Central Hospital in Jazan were included in the analysis. Demographic/clinical data were collected and an Arabic version of the Medical Outcomes 36-Item Short-Form Health Survey (SF-36) questionnaire was used to assess QoL.

**Results:**

SCD patients who hold a university degree reported positive impacts on the following domains of SF-36: physical role function, vitality, emotional well being, social function, pain reduction, and general health (*P* = .002, *P* = .001, *P* = .001, *P* = .003, *P* = .004, and *P* = .001, respectively). In general, patients with fever, skin redness, swelling, or history of blood transfusion tended to impair the health status of the SF-36. A multivariate analysis revealed that patients with a university degree tended to report high scores of physical role functions, emotional role function, and vitality. Patients with regular exercise tend to increase vitality, social function, general health, and reduce pain. Unemployment tends to lessen vitality and worsen pain. On average, pain, social function, and physical function scores tended to worsen in patients with swelling or history of blood transfusion.

**Conclusions:**

This study highlighted that poor education, fever, skin redness, and swelling were negatively associated with specific components of SF-36. SCD patients with a history of blood transfusion found their QoL poorer, whereas regular exercise tended to improve QoL.

## Background

Sickle cell disease (SCD) is a genetic disease that affects humans and is handed down from parents to children. Sickle cell patients report poorer quality of life (QoL) in comparison with the general population and other chronic non-communicable diseases [1–5]. Several international studies have been conducted to examine the QoL of children and adults with SCD [4–12]. There is a lack of research concerning health-related quality of life (HRQoL) in Saudi patients with SCD. A single study conducted in Saudi Arabia examined the QoL of adolescents with SCD [13]. This research has highlighted a number of important factors related to HRQoL. According to the authors, SCD-related complications and socio-demographics were shown to decrease QoL in Saudi adolescents [13]. The study revealed that age, gender, and education were associated with changes in QoL in Saudi adolescents with SCD [13]. However, no study has assessed HRQoL in adult patients with SCD in the entire Arabic world, particularly among Saudi adult populations. SCD is present throughout Saudi Arabia and is particularly common in the Eastern and Southern regions [13]. The frequency of the sickle cell gene in the Hofuf area ranges from 15–25 % of the population in Eastern province of Saudi Arabia [14]. The prevalence of sickle cell disease in Southern region of Saudi Arabia was 10.3 % [15]. Coping with this disease is a very challenging experience, especially for adolescents. Psychosocial problems are common in patients with SCD. Factors such as pain, apprehension, difficulty in fulfilling personal and family responsibility, financial burdens, and diminished cognition must also be encompassed to fully understand what the illness means to an individual [16, 17].

There is a great need to educate the public toward a greater understanding of the connection between pain and their reactions toward the symptoms. Healthcare providers that are less familiar with SCD patients might underestimate the impact of SCD on the quality of life of an individual with SCD (e.g., describing SCD patients as drug dependent [18]). This is due to insufficient knowledge of the severity of SCD and the disease in general. Some providers may not know how to minimize the burden of SCD patients to achieve as normal a life as possible. Therefore, measuring the QoL among adults with SCD might provide significant attention to the health outcome and the degree of severity of the disease. This study represents the first investigation of SCD patients’ QoL in an adult Saudi Arabia population. The current study describes the health-related QoL of Saudi adults with SCD, and examines explanatory predictors that might be associated with poorer QoL. Specifically, we hypothesized that the presence of signs, symptoms, or complications of SCD as reported by fever, skin redness swelling in hands or feet, and blood transfusion are associated with worse HRQoL. The study also investigates the effect of gender, age, physical activity, and other characteristics on HRQoL among adult Saudi patients with SCD. Another purpose of this study was to build predictive models to explain the variation in each of the SF-36 domains.

## Methods

A multicenter, cross-sectional study was conducted in King Fahad Hospital, Hofuf, Eastern region and King Fahad Central Hospital, Jazan, Southern region, Saudi Arabia. This study was approved by the Ministry of Health (IRB Log No. 15-247E), Kingdom of Saudi Arabia, and the King Abdullah International Medical Research Center, Research Protocol - RC12/127/R. Participants in the study were adult patients with sickle cell who were treated in King Fahad Hospital in Hofuf and King Fahad Central Hospital in Jazan. All respondents provided verbal agreement to participate in the study. A total of 629 questionnaires were completed out of the 823 that were distributed, a response rate of 76.4 %. The exclusion criteria included: age < 18 years or patient who can’t read or write. Data about patient demographics and clinical characteristics collected included age, gender, marital status, education, obesity (body mass index of 30 or greater), and employment status. The study participants were asked to report fever (Yes/No), skin redness (Yes/No), swelling in hands or feet (Yes/No), and blood transfusion (Yes/No) during the past three months. In addition to SCD symptoms and complications, the participants were asked to report family history of anemia (Yes/No), presence of other chronic diseases (Yes/No), regular exercise (Yes/No), and whether they are receiving family support (Yes/No).

### Study instrument

The study measured the HRQoL in adult patients with SCD. The Medical Outcomes Study (MOS) short form (SF-36) is a 36-item tool for measuring health status and outcomes from the patients’ perceptions [19]. The English version of the RAND-36 provides a reliable measure of HRQoL, and Cronbach’s alpha values range from 0.78 to 0.93 [19]. This survey has already been translated into Arabic and tested for internal consistency and reliability in a sample of Saudi Arabian citizens [20]. It was found that both the Arabic and English versions of the RAND-36 are equivalent. A previous study used the Arabic version of the RAND-36 to assess health-related quality of life, but this study was conducted in an adolescent population [13]. In our study, adults patients with SCD rated their QoL in terms of their satisfaction and feelings with regard to eight different components, with a total of 36 items addressing eight health concepts: physical function (10 items); physical role health (4 items); emotional role functions (3 items); vitality (4 items); emotional wellbeing (5 items); social function (2 items); bodily pain (2 items); and generalica health perceptions (5 items). Data from SF-36 were scored based on the scoring system reported by RAND Health. Each component has a single summary variable ranging from 0 = poor health to 100 = good health. The internal consistency and reliability of the SF-36 has been investigated in a pilot study of 80 patients with SCD. High internal consistency (Cronbach’s alpha > 0.6) have been reported for physical function (Cronbach’s alpha = 0.81); physical role health (Cronbach’s alpha = 0.84); emotional role functions (Cronbach’s alpha = 0.86); vitality (Cronbach’s alpha = 0.79); emotional wellbeing (Cronbach’s alpha = 0.67); social function (Cronbach’s alpha = 0.67); bodily pain (Cronbach’s alpha = 0.84); and general health (Cronbach’s alpha = 0.60).

### Statistical analyses

The data analysis was performed using IBM SPSS Statistics 20 (SPSS, Chicago, IL).

### Patients’ characteristics

Descriptive statistics such as means and standard deviation (*mean ± SD*) were used to describe the quantitative variables. Frequencies and percentages *n (%)* were used to describe categorical variables (Table 1).

**Table 1:** SCD sample characteristics (*n* =629)

Characteristics	Levels	Number	Percent
Gender	Female	374	59.6
	Male	253	40.4
Older age	No	523	83.1
	Yes	106	16.9
University	No	440	72.5
	Yes	167	27.5
Married	No	342	55.6
	Yes	273	44.4
Employed	No	407	71.4
	Yes	163	28.6
Obese	No	440	90.9
	Yes	44	9.1
Fever	No	246	40.3
	Yes	365	59.7
Skin redness	No	407	66.6
	Yes	204	33.4
Swelling	No	324	53.0
	Yes	287	47.0
History of blood transfusion	No	100	16.1
	Yes	520	83.9
Family history of anemia	No	59	9.6
	Yes	554	90.4
Family support	No	47	7.7
	Yes	560	92.3
Other Chronic disease	No	509	83.3
	Yes	102	16.7
Regular exercise	No	410	66.3
	Yes	208	33.7
Spleen removed	No	508	81.8
	Yes	113	18.2
FS-36 Subscales		Mean	SD
Physical functioning		55.1	23.3
Role limitations due to physical health		35.9	36.0
Role limitations due to emotional problems		40.1	40.5
Vitality		47.8	19.1
Emotional well being		59.3	19.4
Social functioning		60.1	25.0
Pain		47.9	27.6
General health		47.8	15.6

### Bivariate analyses

The impact of patients’ characteristics on health status components (8 components of the SF-36) was evaluated by independent sample t-tests (Tables 2 and 3). Specifically, we compared physical functioning, role limitation due to physical health, role limitations due to emotional problems, vitality, emotional wellbeing, social functioning, bodily pain, and general health perceptions across socio-demographic/ clinical characteristics. In order to account for 15 multiple comparisons, the *p*-values were adjusted using a Bonferroni correction for all the sub-group Analyses. Significance level was evaluated at *P* < 0.05/15 (0.003).

**Table 2:** Differences in health-related quality of life by socio-demographic and clinical characteristics (*N* = 629)

		Physical functioning			Role limitations due to physical health			Role limitations due to emotional problems			Vitality		
		Mean	SD	*P*	Mean	SD	*P*	Mean	SD	*P*	Mean	SD	*P*
Gender	Female	57.0	23.7	0.019	36.5	37.1	0.630	39.1	41.7	0.423	49.0	20.0	0.060
	Male	52.3	22.5		35.0	34.5		41.8	38.5		46.0	17.4	
Older age	No	55.5	23.4	0.472	36.1	35.4	0.767	39.0	39.2	0.218	48.0	18.8	0.484
	Yes	53.5	22.7		34.9	39.1		45.1	46.1		46.6	20.2	
University	No	53.3	22.9	0.004	32.9	34.9	0.002*	38.1	39.7	0.020	45.9	18.3	0.001*
	Yes	59.5	23.5		43.8	37.7		47.1	42.1		53.2	19.4	
Married	No	56.0	23.6	0.401	38.4	34.8	0.078	42.3	39.1	0.183	49.1	18.2	0.061
	Yes	54.3	23.1		33.1	37.6		37.8	42.2		46.1	19.9	
Employed	No	53.4	22.5	0.002*	35.7	36.0	0.379	39.6	39.9	0.283	45.6	18.6	0.001*
	Yes	60.1	23.3		38.7	37.6		43.8	41.8		52.1	19.6	
Obese	No	56.3	23.9	0.086	37.6	36.8	0.309	42.2	40.3	0.766	48.2	19.5	0.188
	Yes	49.7	23.4		31.5	34.9		40.3	42.8		44.2	15.6	
Fever	No	55.9	23.8	0.555	44.6	36.5	0.001*	46.5	41.8	0.002*	53.7	19.2	0.001*
	Yes	54.7	23.1		30.0	34.8		35.8	39.2		43.9	17.9	
Skin redness	No	57.5	22.6	0.001*	40.3	36.4	0.001*	42.7	40.6	0.040	50.3	19.3	0.001*
	Yes	50.5	23.6		27.1	34.1		35.3	40.3		42.9	17.5	
Swelling	No	59.2	22.9	0.001*	41.0	36.9	0.001*	44.8	41.5	0.002*	52.1	20.0	0.001*
	Yes	51.2	22.9		29.7	34.1		34.7	39.0		42.9	16.9	
History of blood transfusion	No	61.7	25.1	0.003*	47.9	37.1	0.001*	50.2	41.5	0.007	53.3	21.1	0.002*
	Yes	53.7	22.8		33.6	35.4		37.9	40.0		46.6	18.5	
Family history of anemia	No	53.3	19.6	0.547	38.5	37.5	0.623	44.2	41.1	0.480	51.7	17.5	0.102
	Yes	55.4	23.5		35.9	36.1		40.1	40.5		47.2	19.1	
Family support	No	57.7	21.3	0.424	35.9	40.0	0.983	45.2	47.2	0.469	46.7	22.2	0.659
	Yes	54.8	23.6		35.8	35.9		39.9	40.0		48.0	18.7	
Other Chronic disease	No	56.0	23.2	0.032	37.6	36.2	0.034	41.4	40.7	0.242	49.0	19.3	0.002*
	Yes	50.4	22.7		29.1	34.6		36.1	39.3		42.4	17.2	
Regular exercise	No	54.1	22.6	0.134	34.8	36.4	0.261	40.3	41.0	0.694	44.7	18.6	0.001*
	Yes	57.3	24.6		38.3	35.1		38.9	39.4		53.5	18.5	
Spleen removed	No	55.1	23.0	0.877	34.7	35.5	0.088	39.5	40.5	0.451	47.7	19.1	0.931
	Yes	54.7	24.9		41.3	38.3		42.8	40.8		47.9	18.8	

*The factor is significant using *Bonferroni correction* cut-off at α/*n* = 0.05/15 = 0.003

**Table 3:** Differences in health-related quality of life by socio-demographic and clinical characteristics (*N* = 629)

		Emotional well being			Social functioning			Pain			General health		
		Mean	SD	*P*	Mean	SD	*P*	Mean	SD	*P*	Mean	SD	*P*
Gender	Female	60.1	19.7	0.241	60.6	25.7	0.510	49.0	28.5	0.268	47.8	15.7	0.876
	Male	58.2	19.1		59.2	24.0		46.5	26.4		48.0	15.6	
Older age	No	59.4	19.1	0.711	59.6	24.8	0.280	48.5	27.3	0.257	47.8	15.8	0.980
	Yes	58.6	21.0		62.5	26.1		45.1	29.4		47.9	15.1	
University	No	57.3	19.1	0.001*	58.4	24.6	0.003*	46.0	26.9	0.004	46.3	15.2	0.001*
	Yes	63.9	19.3		65.4	25.6		53.2	28.5		51.6	16.1	
Married	No	60.7	18.8	0.064	61.8	24.8	0.100	50.0	27.7	0.058	48.6	15.3	0.167
	Yes	57.7	20.2		58.4	24.8		45.7	27.3		46.8	16.2	
Employed	No	58.2	19.5	0.132	58.8	25.2	0.044	46.1	27.7	0.018	47.0	15.2	0.230
	Yes	61.0	19.4		63.6	24.4		52.3	27.7		48.8	16.6	
Obese	No	59.3	19.4	0.836	61.0	24.7	0.099	48.5	27.9	0.455	47.8	16.1	0.395
	Yes	58.6	20.8		54.5	22.1		45.2	24.4		45.5	15.2	
Fever	No	63.1	18.5	0.001*	65.9	25.3	0.001*	54.4	28.4	0.001*	51.5	15.3	0.001*
	Yes	56.9	19.7		56.2	24.0		43.4	26.2		45.6	15.3	
Skin redness	No	61.2	19.7	0.001*	62.6	24.8	0.001*	50.6	28.6	0.001*	50.6	15.3	0.001*
	Yes	55.8	18.4		55.0	24.6		42.6	25.4		42.5	15.1	
Swelling	No	63.0	19.4	0.001*	65.7	24.7	0.001*	56.4	28.8	0.001*	50.9	15.4	0.001*
	Yes	55.1	18.9		53.5	23.9		38.4	23.3		44.5	15.3	
History of blood transfusion	No	62.8	18.7	0.045	69.6	24.1	0.001*	59.0	28.2	0.001*	53.2	15.4	0.001*
	Yes	58.5	19.5		57.7	24.6		45.8	27.2		46.7	15.5	
Family history of anemia	No	63.0	18.3	0.126	66.4	24.7	0.057	55.8	29.0	0.023	48.9	14.6	0.610
	Yes	58.8	19.4		59.5	24.8		47.0	27.3		47.7	15.7	
Family support	No	56.9	20.6	0.328	60.6	23.8	0.896	53.9	28.4	0.126	51.5	15.3	0.118
	Yes	59.8	19.2		60.0	25.2		47.4	27.5		47.6	15.7	
Other Chronic disease	No	60.8	19.1	0.001*	61.7	24.5	0.002*	49.1	27.9	0.088	48.4	15.9	0.056
	Yes	52.5	19.8		53.0	24.9		43.8	25.1		44.9	14.3	
Regular exercise	No	57.5	19.3	0.002*	58.5	24.5	0.031	45.8	27.0	0.011	45.9	15.5	0.001*
	Yes	62.8	19.3		63.2	25.4		51.8	28.5		51.0	15.0	
Spleen removed	No	59.9	19.3	0.079	60.7	25.0	0.277	48.0	27.5	0.981	48.1	15.6	0.477
	Yes	56.2	20.3		57.8	23.9		48.0	28.2		46.9	16.0	

*The factor is significant using *Bonferroni correction* cut-off at α/*n* = 0.05/15 = 0.003

### Regression analyses

Multiple linear regression models were used to identify important predictors of each health status component (Table 4). In multiple linear regression analyses, *P* < 0.05 was considered significant.

**Table 4:** Multiple regression showing predictors of health-related quality of life in patients with SCD

	Physical functioning		Role limitations due to physical health		Role limitations due to emotional problems		Vitality		Emotional well being		Social functioning		Pain		General health	
Predictors	B	*P*	B	*P*	B	*P*	B	*P*	B	*P*	B	*P*	B	*P*	B	*P*
(Constant)	76.3		61.7		45.3		50.6		74.6		77.5		77.3		59.6	
Gender	−3.1	0.278	−0.2	0.964	1.7	0.714	1.1	0.619	−0.6	0.801	1.9	0.503	4.6	0.126	2.9	0.113
Age	−0.2	0.327	0.2	0.519	0.6	0.020*	0.3	0.036*	0.0	0.772	0.2	0.202	−0.1	0.704	0.0	0.834
University	4.9	0.077	10.9	0.009*	13.9	0.003*	5.3	0.013*	3.7	0.102	2.6	0.359	4.1	0.179	3.4	0.060
Married	−3.8	0.191	−8.4	0.056	−15.7	0.001*	−6.9	0.002*	−5.2	0.029*	−6.1	0.036*	−5.7	0.069	−2.8	0.146
Employed	5.1	0.086	1.9	0.673	0.2	0.960	7.3	0.001*	3.5	0.139	3.9	0.190	6.8	0.036*	1.8	0.347
Obese	−1.5	0.717	−3.7	0.555	3.0	0.667	−2.8	0.366	2.1	0.544	−6.1	0.143	−1.4	0.750	−1.0	0.711
Fever	3.2	0.241	−8.7	0.032*	−5.6	0.212	−6.6	0.001*	−3.0	0.162	−5.2	0.054	−4.2	0.148	−1.9	0.292
Skin redness	−5.7	0.033*	−10.9	0.008*	−5.5	0.226	−2.5	0.220	−1.9	0.382	−3.5	0.203	−2.1	0.472	−4.3	0.016*
Swelling	−8.1	0.003*	−6.0	0.131	−7.1	0.108	−4.2	0.036*	−3.4	0.111	−8.4	0.002*	−14.4	0.001*	−4.3	0.013*
Blood transfusion	−6.7	0.046*	−9.8	0.052	−12.9	0.022*	−1.6	0.524	−0.9	0.726	−7.4	0.026*	−8.3	0.024*	−5.9	0.007*
Family history of anemia	−2.8	0.620	−8.6	0.291	2.4	0.788	−5.2	0.208	−7.0	0.119	−6.2	0.239	−8.8	0.125	−2.1	0.564
Family support	−3.7	0.381	−3.2	0.621	−3.3	0.652	1.1	0.730	−1.1	0.754	−2.1	0.638	−7.6	0.107	−4.0	0.160
Other Chronic disease	−4.0	0.293	−5.5	0.328	−1.0	0.867	−6.7	0.019*	−9.7	0.001*	−10.1	0.008*	−3.7	0.369	−0.7	0.786
Regular exercise	0.0	0.988	4.7	0.239	−1.3	0.764	5.6	0.005*	1.7	0.433	6.1	0.024*	7.5	0.009*	5.2	0.003*
Spleen removed	4.2	0.192	8.1	0.090	11.8	0.027*	0.9	0.716	−5.1	0.048*	−3.1	0.326	2.2	0.514	2.3	0.267
F-value	3.2		3.6		2.8		5.4		3		4.3		5.7		4.1	
*P*-value	0.001		0.001		0.001		0.001		0.001		0.001		0.001		0.001	
R Square	0.12		0.13		0.10		0.18		0.11		0.15		0.18		0.15	
R	0.35		0.36		0.32		0.42		0.33		0.39		0.43		0.38	

* Controlling for other predictors in model, predictor is significant at α = 0.05

## Results

### Patients’ characteristics

Characteristics of the patients are presented in Table 1. Of 629 SCD patients included in the study, 40.4 % were males. The mean age of the patients was 28.8 (SD ± 9.1 years) with range between 18 and 75 years. A total of 273 patients (44.4 %) were married, 407 (71.4 %) were unemployed, and 44 (9.1 %) were obese. Among 629 SCD patients, 59.7 % had fever, 33.4 % had skin redness, and 47 % had swelling. The majority (83.9 %) of the patients had a history of blood transfusion, (90.4 %) had a family history of anemia, 92.3 % reported that they receive family support, and 16.7 % reported having chronic disease other than anemia. Of 366 SCD patients, 75 (18.2 %) had their spleen removed.

### Bivariate analyses

The bivariate impacts of patients’ characteristics on SF-36 subscales are presented in (Tables 2 and 3). The average physical function score was 55.1 (SD = 23.3). Unemployed patients had a significantly lower physical function than employed patients, as did patients who had a history of skin redness, swelling, and blood transfusions. Patients had a mean physical role function of 35.9 (SD = 36). Table 2 displays the bivariate associations between patients’ characteristics and physical role functions. Patients with a university degree had better physical role function than in patients with no university degree. Conversely, physical role function was considerably worse among patients with fever, skin redness, swelling, and history of blood transfusion. The mean emotional role function of patients was 40.1 (SD = 40.5). The emotional role function was considerably worse in those with fever and swelling. The vitality component had a mean of 47.8 (SD = 19.1). Vitality scores were somewhat higher for patients with a university degree. Unemployed patients had significantly poorer vitality. Furthermore, patients with fever, skin redness, swelling, history of blood transfusion, presence of other chronic diseases, and no regular exercise had poorer scores in vitality.

The mean of emotional wellbeing was 59.3 (SD = 19.4). Patients with a university degree had significantly better emotional wellbeing than patients with no university degree. Our bivariate analyses identified fever, skin redness, swelling, no regular exercise, and the presence of other chronic diseases as demonstrating significantly poorer emotional wellbeing. The mean social function component was 60.1 (SD = 25). Patients with a university degree had significantly better social function than patients with no university degree. The clinical predictors that were significantly associated with reduced social function were fever, skin redness, swelling, blood transfusion, and presence of chronic disease other than SCD.

**Fig. 1:**
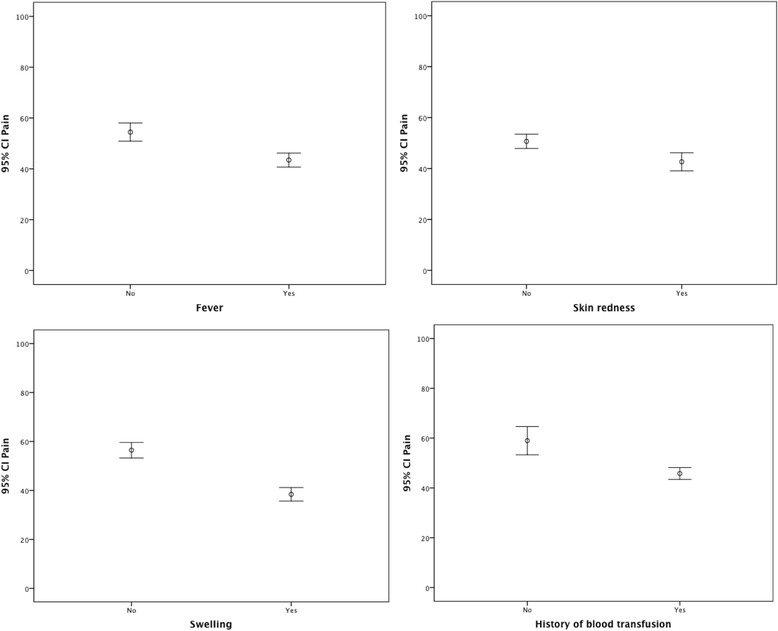
Error bar charts, impact of SCD complications on bodily pain ratings. The higher the score, the less pain

The mean bodily pain domain was 47.9 (SD = 27.6). Bivariate analyses showed that patients who hold a university degree had significantly less pain than patients with no university degree. However, more pain was associated with fever, skin redness, swelling, and history of blood transfusion (see Table 3 and Fig. 1). General health components had a mean of 47.8 (SD = 15.6). Better general health was significantly associated with a university degree. Worse general health was significantly associated with fever, skin redness, swelling, history of blood transfusion, and no regular exercise.

### Regression analyses

Table 4 shows the results of the multiple linear regression models. Controlling for all 15 predictors, patients who hold a university degree tend to report higher scores on the physical role function, emotional role function, and vitality relative to patients with no university degree (increase in SF-36 scores of 10.9, 13.9, and 5.3, respectively). Married patients tend to report low scores on vitality, emotional wellbeing, and social functioning (decrease in SF-36 scores of 6.9, 5.2, 6.1, respectively) than unmarried patients. Employed patients tend to report better scores on vitality and pain (increase in SF-36 scores of 7.3 and 6.8, respectively) than unemployed patients.

Based on the multiple linear regression results in Table 4, patients who had a history of blood transfusion tend to have poor physical function, emotional role function, social function, general health, and worse pain, (decrease in SF-36 scores of 6.7, 12.9, 7.4, 5.9, 8.3, respectively) relative to patients who had no history of blood transfusion. Patients with a fever tend to have a negative impact on physical role function and vitality (decrease in SF-36 scores of 8.7 and 6.6, respectively) relative patients with no fever. Patients with swelling tend score less on physical function, vitality, social function, pain, and general health (decrease in SF-36 scores of 8.1, 4.2, 8.4, 14.4, and 4.3, respectively) relative to patients with no swelling. Patients with skin redness tend to have scores on physical function, physical role function, general health that are (decrease in SF-36 scores of 5.7,10.9, and 4.3, respectively) lower than patients with no skin redness. Regular exercise tends to increase vitality, social function, and general health and reduce pain (increase in SF-36 scores of 5.6, 6.1, 5.2, and 7.5, respectively).

## Discussion

Research on health-related quality of life among patients with SCD in Saudi Arabia has not received the attention it deserves. Only one study assessed HRQoL in a sample of Saudi adolescents with SCD [13]. The current study represents the first investigation to assess the health-related quality of life in adult Saudi patients with SCD. There are no data about QoL in the Saudi general population to make comparisons with our SCD sample. A study was conducted at King Khalid University to describe the QoL among university students [21]. The university students demonstrated significantly better physical functioning (79.37 vs. 55.1) and general health (70.47 vs. 47.8) as compared with our SCD patients. Surprisingly, the bodily pain among the university students was even worse as compared with SCD patients (38.47 vs. 47.9) in our sample. However, a university sample is not the most appropriate sample to represent the general population.

A study from the United States [1] reported that the average bodily pain among patients with SCD was 47.4 which was consistent with our study (47.9). Saudi adolescents with SCD reported worse bodily pain compared to adults with SCD studied (33.74 [13] vs. 47.9 in the current investigation and 47.4 in McClish et al., [1]). Also, Saudi adolescents with SCD reported poorer social functioning than adults with SCD (44.73 [13] vs. 61.1 in the current investigation and 63.5 in McClish et al., [1]). This indicates that the impact of SCD on pain and social functioning for adolescents tended to be greater than that of adults. Previous study found that there were significant differences in QoL between men and women with SCD [1, 13, 21]. According to our study we found that men and women did not differ in any of the eight perceived health domains. There was no evidence of difference in HRQoL between young and old age groups. The current study revealed that HRQoL among SCD patients was observed significantly better among patients with a university degree as compared to patients with no university degree.

In our study, the most common clinical conditions observed in patients with SCD were fever, presented in (59.7 %) of the sample, history of blood transfusion (83.9 %), and family history of anemia (90.4 %). The bivariate analyses revealed that clinical conditions such as fever, skin redness, swelling, and history of blood transfusion were significant predictors of several perceived health domains. These clinical conditions tend to worsen SCD patient quality of life.

The results of multiple linear regression confirmed that swelling was negatively related to physical function, vitality, social function, bodily pain, and general health, whereas having a university degree was positively related with physical role function, emotional role function, and vitality. Regular exercise positively impacted general health, bodily pain, social functioning, and vitality.

The current study has highlighted certain factors that are necessary to manage in order to deal with the pain and improve QoL in patients with SCD. The research emphasizes the importance of regular exercise habits to control the disease burden by developing physical exercise/ training programs for people living with SCD. Proper education about the disease will play a major role in helping families understand what their patient is going through. It is essential to assess SCD patients’ QoL to effectively manage the physical pain caused by the disease. The presence of signs, symptoms, or complications of SCD seems to have a negative impact on QoL among Saudi SCD patients. We observed worse pain, physical function, and social function in Saudi SCD patients with skin redness or swelling. It is important for hematologists to evaluate patients’ QoL as part of the treatment and management of SCD. Our study suggests screening for SCD complications among patients with SCD and providing appropriate counseling to avoid worse health outcomes.

Interventional studies, such as one to assess the role of regular exercise to prevent poor QoL related to SCD, are needed. It is essential for the public as well as a patient’s close family and relatives to know that, although SCD is a serious condition, those with the disease can essentially lead fairly normal lives provided that they follow a regular exercise program. Several limitations were noted in our study. This is an observational study and only shows correlations, but that doesn’t necessarily imply causation. The comparison with the averages reported from other studies must to be taken with caution, given the different methodological issues and the subjectivity of the surveys. However, this is the first study in terms of assessing health-related quality of life among adult patients with SCD in Saudi Arabia and the entire Arabic world. The results of the study may be useful not only to SCD patients but also to physicians who assess signs or symptoms for these patients in their medical practice.

## Conclusion

Regular clinical symptoms checks are important. Clinical symptoms such as fever, skin redness, swelling, and history of blood transfusion had considerable negative impact on several SF-36 domains. Regular exercise tends to lower pain and improves social functions and vitality. Interventional study is needed to evaluate the effectiveness of exercise on quality of life outcomes and pain management in SCD patients.
